# Mechanisms involved in the triggering of neutrophil extracellular traps (NETs) by *Candida glabrata* during planktonic and biofilm growth

**DOI:** 10.1038/s41598-017-13588-6

**Published:** 2017-10-12

**Authors:** Chad J. Johnson, John F. Kernien, Amanda R. Hoyer, Jeniel E. Nett

**Affiliations:** 1 Department of Medicine, University of Wisconsin, Madison, WI United States of America; 2 Department of Medical Microbiology and Immunology, University of Wisconsin, Madison, WI United States of America

## Abstract

*Candida* spp. adhere to medical devices, such as catheters, forming drug-tolerant biofilms that resist killing by the immune system. Little is known about how *C*. *glabrata*, an emerging pathogen, resists attack by phagocytes. Here we show that upon encounter with planktonic (non-biofilm) *C*. *glabrata*, human neutrophils initially phagocytose the yeast and subsequently release neutrophil extracellular traps (NETs), complexes of DNA, histones, and proteins capable of inhibiting fungal growth and dissemination. When exposed to *C*. *glabrata* biofilms, neutrophils also release NETs, but significantly fewer than in response to planktonic cells. Impaired killing of biofilm parallels the decrease in NET production. Compared to biofilm, neutrophils generate higher levels of reactive oxygen species (ROS) when presented with planktonic organisms, and pharmacologic inhibition of NADPH-oxidase partially impairs NET production. In contrast, inhibition of phagocytosis nearly completely blocks NET release to both biofilm and planktonic organisms. Imaging of the host response to *C*. *glabrata* in a rat vascular model of infection supports a role for NET release *in vivo*. Taken together, these findings show that *C*. *glabrata* triggers NET release. The diminished NET response to *C*. *glabrata* biofilms likely contributes to the resilience of these structured communities to host defenses.

## Introduction

The most common nosocomial fungal pathogen, *Candida*, causes disseminated, invasive disease with mortality near 35%^1–3^. Patients treated with immunosuppressive medications, receiving antibiotics, or residing in intensive care units are at particularly high risk for infection^4^. In addition, indispensable medical devices, such as vascular catheters, allow *Candida* to form resilient, surface-associated biofilm communities that tolerate antifungal drugs and resist host defenses^5–10^. Nearly 80% of patients with invasive candidiasis have implanted medical devices^2^. The fungal biofilms associated with these devices are notoriously difficult to eradicate clinically, and studies are just beginning to shed light on how they survive immune attack^5–13^. *C*. *albicans* has served as the model pathogen for the majority of these investigations.

Neutrophils are a critical first line of defense against fungal pathogens, including *Candida* ^2,14–18^. In response to planktonic, or non-biofilm *C*. *albicans*, they release neutrophil extracellular traps (NETs), which are webs of DNA, histones, and proteins with antifungal activity^19–22^. These structures can prevent pathogen dissemination and kill organisms too large to be phagocytosed^19,20^. However, a recent investigation revealed that neutrophils fail to release NETs in response to biofilms produced by *C*. *albicans* ^12^. This explains, in part, why neutrophils are ineffective at controlling *C*. *albicans* device-associated biofilm infections. The impaired neutrophil function was linked to the biofilm matrix, the fungal-produced extracellular material that coheres cells within the biofilm and protects them from external insults^12,23^. The composition of this material varies significantly among *Candida* spp. and it remains a mystery if other species employ this mechanism to resist killing by neutrophils^24–27^.

Historically, *C*. *albicans* has accounted for the majority of invasive candidiasis. However, there has been a global shift and emergence of non-*albicans Candida spp*., which now account for 55% of disease^3^. Of these, *C*. *glabrata* is frequently the most prevalent, particularly in Europe and North America^18,28^. This emerging pathogen poses obstacles to treatment, including increased intrinsic resistance to commonly used antifungals, such as azoles, and the rapid emergence of drug resistance during treatment^4,29^. The mortality for invasive *C*. *glabrata* infection is astonishingly high, even greater than that for *C*. *albicans* ^18,30^.

Like *C*. *albicans*, *C*. *glabrata* forms biofilms on medical devices and mucosal surfaces^6,31,32^. However, *C*. *glabrata* and *C*. *albicans* vary genetically and morphologically^29,33^. The genome of *C*. *albicans* is diploid, whereas *C*. *glabrata* is haploid and phylogenetically closer to *Saccharomyces cerevisiae*, or baker’s yeast. *C*. *glabrata* grows strictly as a relatively small yeast (1–4 µm), while *C*. *albicans* produces larger yeast forms (4–7 µm), as well as filamentous forms (pseudohyphae and hyphae), which serve as the more potent stimuli for NET formation^20,34^. In the current study, we investigate the neutrophil response to *C*. *glabrata* during planktonic and biofilm growth and identify stark differences compared to *C*. *albicans*.

## Results

### Neutrophils release NETs in response to planktonic *C*. *glabrata*

**Figure 1:**
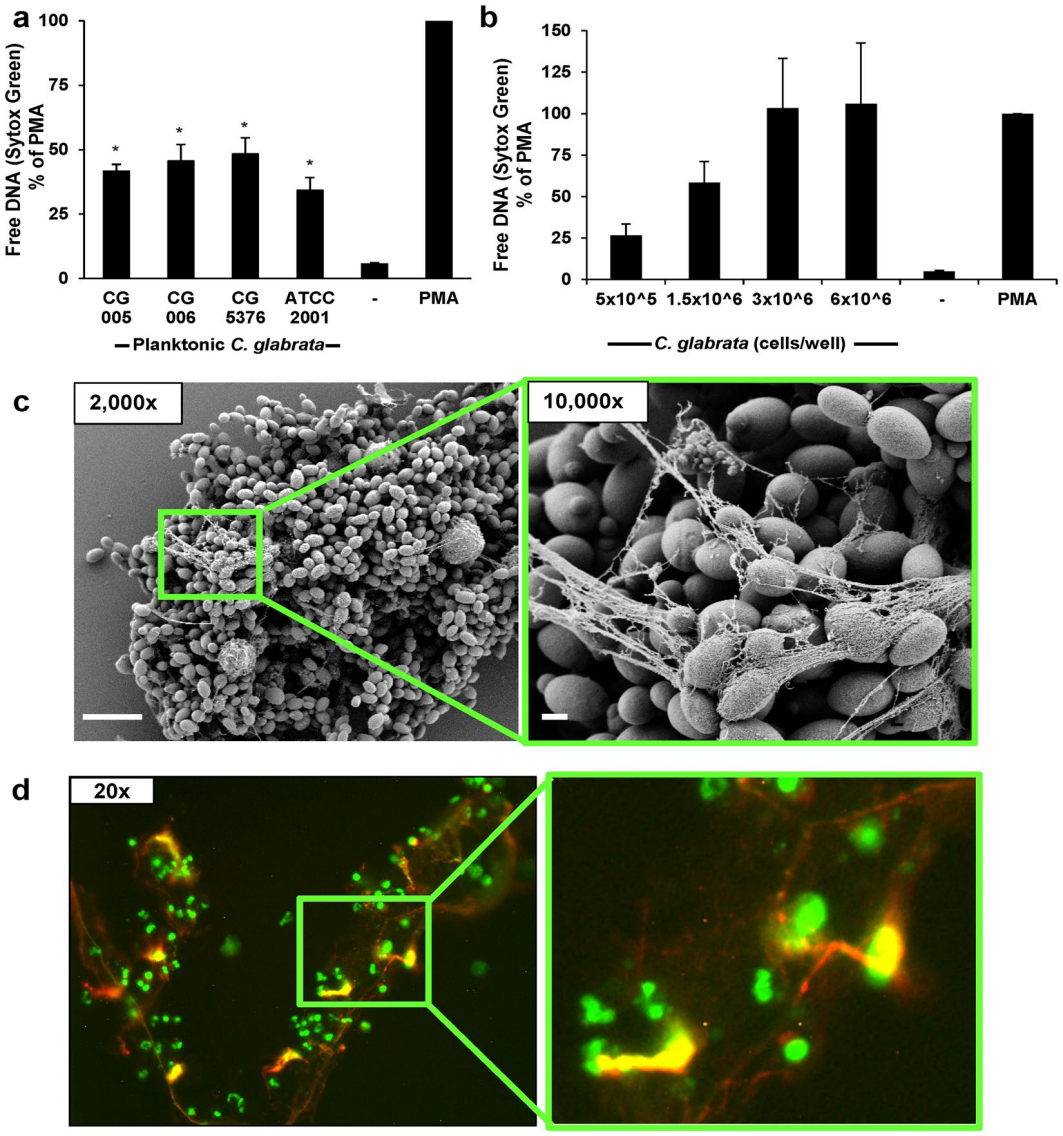
Planktonic *C*. *glabrata* induce release of NETs. (**a**) Human neutrophils were exposed to four strains of planktonic *C*. *glabrata* (1.5 × 10^6^ cells/well) for 4 h. NET release was estimated by Sytox Green detection of free DNA. Results were normalized for the positive control, PMA, and data from 5 experiments performed in triplicate were combined. Neutrophil responses to *Candida* were analyzed by ANOVA with pairwise comparison to the untreated neutrophil control, *P < *0*.*05*, *SEM shown*. (**b**) Various concentrations of planktonic *C*. *glabrata* (CG 006) were co-cultured with human neutrophils for 4 h and NET release was estimated using Sytox Green. Results were normalized for the positive control, PMA, and data from 5 experiments performed in triplicate were combined, *SEM shown*. (**c**) Neutrophil interactions with planktonic *C*. *glabrata* at 4 h were imaged with scanning electron microscopy. Measurement bars represent 10 µm and 1 µm for 2,000x and 10,000x images, respectively. (**d**) Following co-culture with *C*. *glabrata* for 4 h, the neutrophil response was visualized by immunofluorescence using an anti-citrullinated H4 antibody (red) and Sytox Green staining of DNA (green).

Prior investigations have demonstrated NET release to hyphal, but not yeast forms of *C*. *albicans* ^20^. As *C*. *glabrata* does not filament to form hyphae, our initial experiments examined the neutrophil response to planktonic *C*. *glabrata* with the inclusion of strains collected from the bloodstream, vagina, and gastrointestinal tract of patients^35,36^. To estimate NET release, we utilized Sytox Green, a cell-impermeable dye that fluoresces upon DNA binding^12,37^. After co-culture with human neutrophils for 4 h, each *C*. *glabrata* strain triggered fluorescence, consistent with the production of NETs (Fig. 1a). At a concentration of 1.5 × 10^6^ cells/well, each strain elicited similar fluorescence. These values ranged from approximately 35–50% of the levels measured for the positive control, phorbol myristate acetate (PMA), a potent stimulus for NET production^38^. Given the similarity among the neutrophil responses to the strains, we selected a single strain (CG 006) for additional planktonic studies. We next examined the neutrophil response to a variety of *C*. *glabrata* concentrations, representing E:T ranging from 2:5 to 1:30. With increasing *C*. *glabrata* concentrations, free DNA increased to the levels of the positive control (PMA) (Fig. 1b).

To determine if the Sytox Green staining of free DNA represented NET release, we visualized neutrophil-*Candida* interactions by scanning electron microscopy (Fig. 1c). After a 4 h co-culture, we observed fibrillary material extruding from neutrophils and web-like structures coating the planktonic *C*. *glabrata*, consistent with the release of NETs. To further evaluate for NET formation, we utilized immunofluorescence labeling of citrullinated histones, a modification frequently present in NETs^39^ (Fig. 1d and Supplementary Fig. 1). Fluorescent microscopy revealed citrullinated histones (red) co-localizing to the extracellular DNA associated with the neutrophils (green). In this assay, the neutrophils also stained by Sytox Green, due to the membrane permeabilization required for sample processing. Together, these studies reveal extrusion of NETs in response to planktonic *C*. *glabrata*.

### Planktonic *C*. *glabrata* induce rapid NETosis

**Figure 2:**
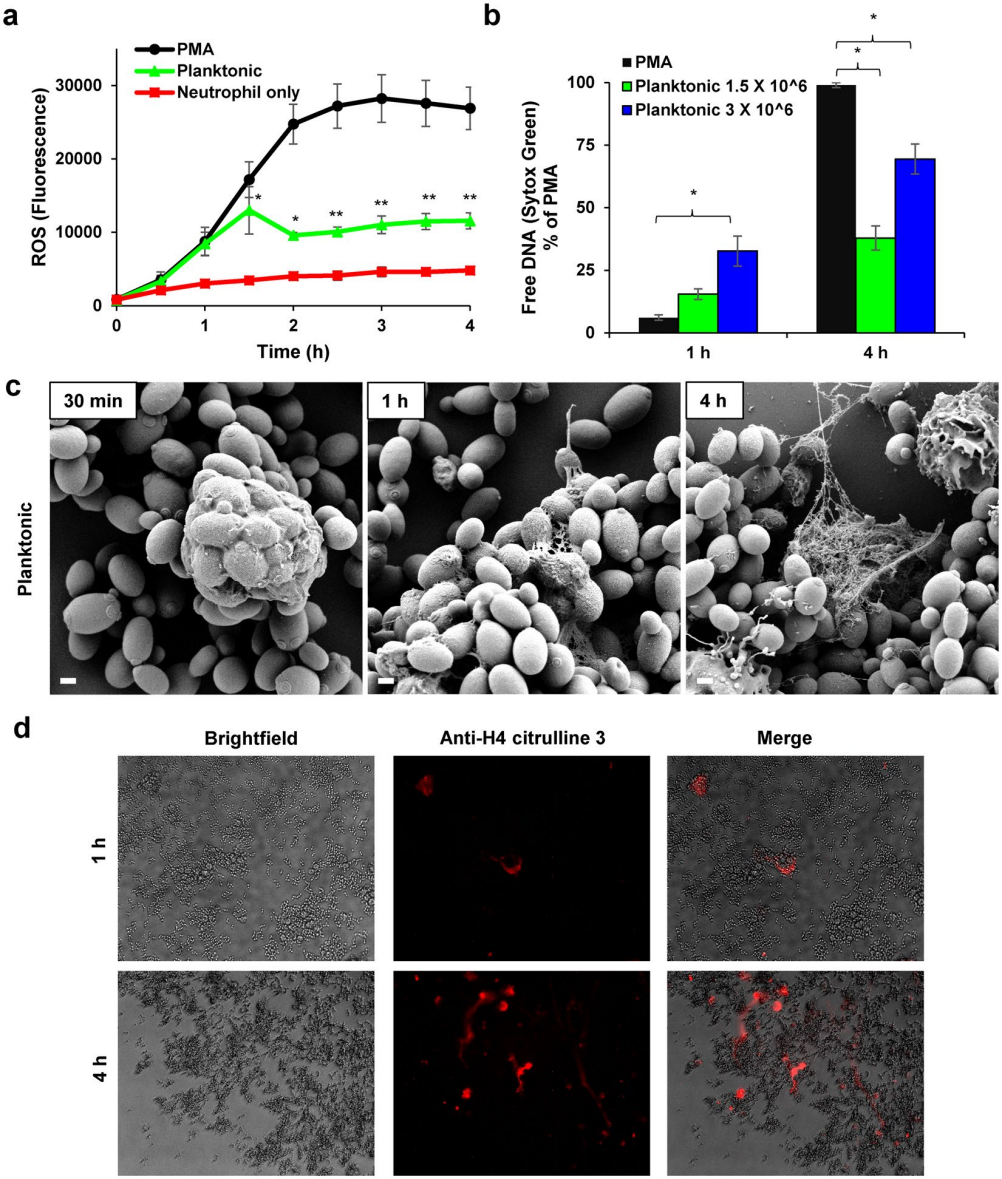
Mechanism of NET release to planktonic *C*. *glabrata*. (**a**) Production of ROS in response to *C*. *glabrata* (CG 006 at 3 × 10^6^ cells/well) was measured by fluorescence after neutrophils were pre-stained with oxidative stress indicator CM-DCF and co-cultured with *C*. *glabrata* over 4 h. The mean and SEM of 4 experiments performed in triplicate on 4 occasions are shown. Data for each time point were analyzed by ANOVA (*P < *0*.*05*), with pairwise comparisons using the Holm-Sidak method (**P < *0*.*05* for *C*. *glabrata* v. PMA). (**b**) Neutrophils were co-cultured with planktonic *C*. *glabrata* for various time points and NET production was estimated by Sytox Green. Results were normalized to the positive control, PMA, and data from 4 experiments performed in triplicate were combined, *SEM shown*. Data for each time point were analyzed by ANOVA (*P < *0*.*05* for pairwise comparison by Holm-Sidak). (**c**) The neutrophil response to planktonic *C*. *glabrata* was imaged with scanning electron microscopy at various time points over 4 h. Measurement bars represent 1 µm for 10,000x images. (**d**) Following co-culture with *C*. *glabrata* for 1 h or 4 h, samples were immunolabeled with an anti-citrullinated H4 antibody and a fluorescently labeled (DyLight) secondary antibody and examined with brightfield microscopy and fluorescent (565/620 nm) microscopy.

Prior investigations have demonstrated NET release to pathogens through distinct pathways, which vary in their timing of release, capacity for microbial killing, dependence on reactive oxygen species (ROS), and involvement of histone citrullination^34,38–43^. To further characterize NET release in response to planktonic *C*. *glabrata*, we performed several complementary experiments. We first considered the involvement of ROS in NET release and analyzed the generation of ROS by neutrophils pre-stained with a free radical sensor (CM-H2DCFDA). Over the course of 90 min, the ROS production in response to *C*. *glabrata* mirrored that of PMA, a strong inducer of ROS-dependent NETosis (Fig. 2a)^44^. Of note, the pattern of ROS production in response to *C*. *glabrata* and PMA differed after 90 min, with ROS production plateauing for *C*. *glabrata*, but continuing to rise for PMA, ultimately reaching 2-fold higher levels. These findings suggest that NET release to planktonic *C*. *glabrata* may involve ROS-dependent pathways.

Our next experiments examined the timing of NET release to planktonic *C*. *glabrata*. In contrast to NETs produced through ROS-dependent pathways, those formed independent of ROS are termed “rapid” NETs for their earlier release^40,41^. Therefore, to further explore NET release to *C*. *glabrata*, we examined the kinetics of NET formation using a Sytox Green assay. PMA, an inducer of the ROS-dependent pathway, did not generate NETs at the early (1 h) time point, with free DNA levels similar to neutrophil-only controls (Figs 1a,b and 2b). However, at the later time point (4 h), PMA induced free DNA levels over 10-fold higher. In contrast, co-culture with *C*. *glabrata* (1.5 × 10^6^ cells/well) induced a more rapid rise of free DNA, but the levels reached less than 40% of the PMA-treated cells (Fig. 2b). The pattern of NET release to a higher concentration of *C*. *glabrata* (3 × 10^6^ cells/well) paralleled that of the lower concentration, augmenting the more rapid NET release. At 1 h, free DNA levels had already reached approximately half of the total observed in response to *C*. *glabrata* at 4 h. The early elevation of free DNA in response to *C*. *glabrata* suggests the involvement of rapid NETosis.

We further analyzed neutrophil interactions with planktonic *C*. *glabrata* over time with scanning electron microscopy (Fig. 2c). After a 30 min co-culture, neutrophils had phagocytosed numerous yeast cells, often multiple per neutrophil. At 1 h, fibrillar material was beginning to extrude from many of the neutrophils. After a 4 h co-culture, webs of material encased the groups of *C*. *glabrata*, consistent with the release of NETs. Immunofluorescent imaging for citrullinated histones mirrored the scanning electron microscopy findings of early NET formation. Citrullination of histones was observed within the first hour of co-culture with *C*. *glabrata* and increased over the course of the 4 h experiment (Fig. 2d). High levels of histone citrullination during NETosis have also been linked with a more rapid release of NETs, suggesting the involvement of a similar pathway in response to *C*. *glabrata* ^42^.

### *C*. *glabrata* biofilms induce NETs

**Figure 3:**
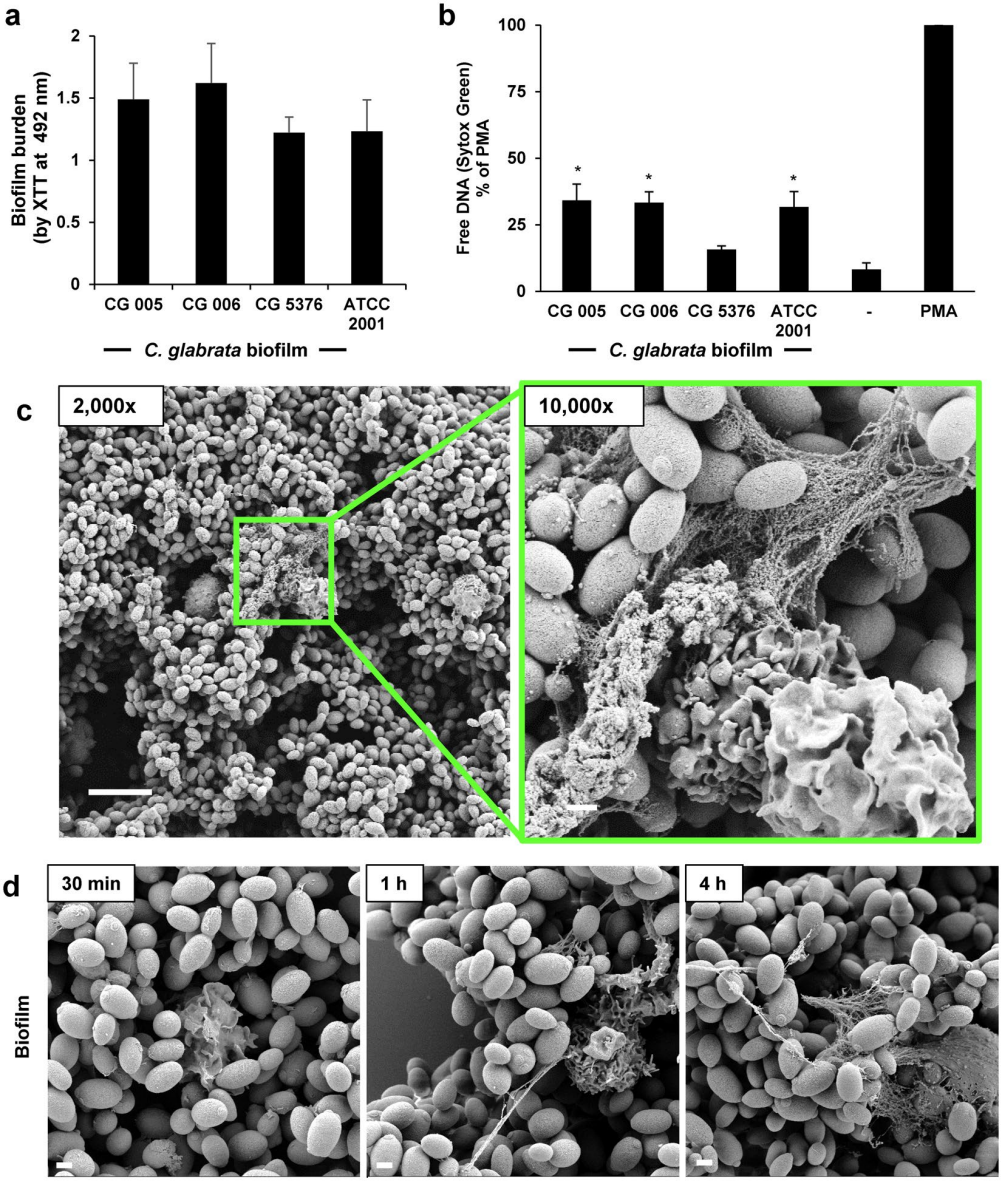
Biofilms formed by *C*. *glabrata* elicit NET release. (**a**) The biofilm-forming capacity of *C*. *glabrata* isolates was estimated by XTT assay after 24 h of growth. Assays were performed in triplicate on 3 days and representative data is shown with SD. (**b**) *C*. *glabrata* biofilms (24 h) were co-cultured with human neutrophils and NET release was estimated by Sytox Green detection of free DNA. Results were normalized to the positive control, PMA, and data from 5 experiments performed in triplicate were combined. Neutrophil responses to *Candida* were analyzed by ANOVA with pairwise comparison to the untreated neutrophil control, *P < *0*.*05*, *SEM shown*. (**c**) Neutrophil interactions with *C*. *glabrata* at 4 h were imaged with scanning electron microscopy. Measurement bars represent 10 µm and 1 µm for 2,000x and 10,000x images, respectively. (**d**) The neutrophil response to *C*. *glabrata* biofilms was imaged with scanning electron microscopy at various time points over 4 h. Measurement bars represent 1 µm for 10,000x images.

During biofilm growth, *C*. *albicans* fails to elicit the release of NETs, promoting survival upon neutrophil attack^12^. Our next studies asked if *C*. *glabrata* employs a similar strategy to impair neutrophil function. As *C*. *glabrata* lacks the capacity for the generation of hyphal or pseudohyphal forms, biofilms formed on coverslips and imaged by scanning electron microscopy revealed mats of adherent yeast (Supplementary Fig. 2). In a microtiter plate model, each of the four clinical isolates similarly formed biofilms with viable burden estimated by XTT metabolic activity after 24 h of growth (Fig. 3a). The biofilm viable burdens were equivalent to planktonic cultures of approximately 3 × 10^6^ to 6 × 10^6^ cells/well (Supplementary Fig. 3). To examine the biofilm-neutrophil interactions, we exposed neutrophils to biofilms for 4 h and measured free DNA with a Sytox Green assay (Fig. 3b). Each of the isolates elicited free DNA, with levels reaching approximately 20–40% of the PMA positive control, consistent with NET release. Using a coverslip model of biofilm formation, we next imaged biofilm-neutrophil interactions by scanning electron microscopy. Following an adherence period and 24 h growth period, *C*. *glabrata* formed a dense biofilm on the coverslip, consisting of clusters of adherent ovoid yeast (Supplementary Fig. 2). After a 4 h co-culture, web-like structures were seen protruding from many of the neutrophils, indicating NET formation (Fig. 3c). Like the neutrophil response to planktonic *C*. *glabrata* (Fig. 2), NET release appeared to progress over the 4 h time course (Fig. 3d).

### *C*. *glabrata* biofilms resist neutrophil killing by modulating NET release

**Figure 4:**
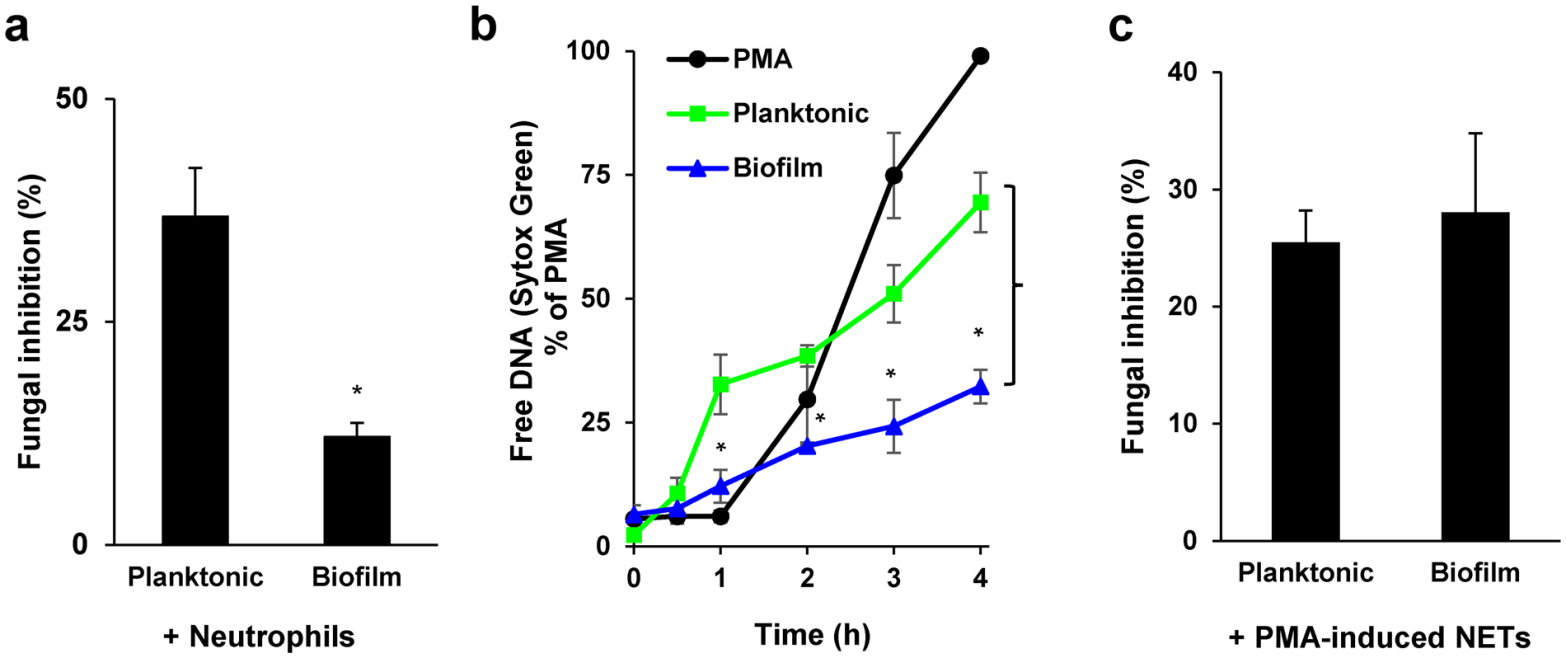
Comparison of neutrophil responses to *C*. *glabrata* during biofilm and planktonic growth. (**a**) Planktonic and biofilm *C*. *glabrata* were co-cultured with human neutrophils at an effector:target ratio of 1:2 for 4 h and fungal inhibition was estimated by an XTT assay. Results were normalized to the no neutrophil controls, and data from 3 experiments performed in triplicate were combined. Statistical significance was determined using a two-tailed Student’s t-test assuming unequal variances, *P < *0*.*05*, *SEM shown*. (**b**) Neutrophils were co-cultured with *C*. *glabrata* for 4 h and NET release was estimated by Sytox Green staining of free DNA at various time points. Results were normalized for the positive control, PMA, and data from 4 experiments performed in triplicate were combined. The statistical significance for NET release to biofilm and planktonic *C*. *glabrata* were analyzed for each time point using a two-tailed Student’s t-test assuming unequal variances, *P < *0*.*05*, *SEM shown*. (**c**) NETs were induced by incubation with PMA for 1.5 h prior to addition to *C*. *glabrata*. After 10 min, fungal inhibition was estimated by an XTT assay. Results were normalized to the no neutrophil controls, and data from 4 experiments performed in triplicate were combined. Statistical significance was determined using a two-tailed Student’s t-test assuming unequal variances, *P < *0*.*05*, *SEM shown*.

Prior investigations have demonstrated that biofilms formed by *C*. *albicans* and *C*. *parapsilosis* resist neutrophil killing^9,11,12,45^. To investigate a similar survival advantage for *C*. *glabrata* biofilms, we utilized a killing assay which measures fungal inhibition by neutrophils with an XTT assay^12^. The activity of neutrophils against biofilms was compared to a similar burden of planktonic organisms (3 × 10^6^ cells/well), as estimated for CG 006 (Supplementary Fig. 3)^12^. A 4 h exposure to neutrophils reduced the viable burden of biofilm by 12% (Fig. 4a). Time course analysis revealed impaired killing by neutrophils after as little as 6 h of biofilm growth (Supplementary Fig. 4). In contrast, inhibition of planktonic *C*. *glabrata* was 3-fold higher (36%). These results suggest that biofilm formation by *C*. *glabrata* is a protective mechanism to evade killing by neutrophils.

One mechanism to account for the resistance of *C*. *glabrata* biofilms to neutrophil killing is a diminished or delayed production of NETs. Utilizing Sytox Green assays as a measure of NET release, we compared the neutrophil response to biofilm and planktonic *C*. *glabrata*. By 1 h, a significantly higher level of NET formation was observed in response to planktonic *C*. *glabrata*, with free DNA levels approximately 35% of the maximum observed for the PMA control (Fig. 4b). The levels continued to rise over time, ultimately reaching near 70% of the control, consistent with a rapid and robust production of NETs. In contrast, NETs were released more slowly in response to biofilms, with free DNA levels near the baseline at 1 h, less than one third of the levels measured for planktonic organisms. After 4 h, the NET response to biofilm was less than half of that observed in response to planktonic organisms. This time course analysis indicates that delayed and impaired NET release is a potential mechanism of innate immune evasion for *C*. *glabrata* biofilms.

We next asked if production of NETs is a successful neutrophil strategy to combat *C*. *glabrata* during planktonic or biofilm growth. To address this question, we induced NETs by PMA treatment of neutrophils for 1.5 h. Following application to *C*. *glabrata*, we measured fungal inhibition with an XTT metabolic assay. A 10 min exposure to PMA-induced NETs similarly inhibited both biofilm and planktonic *C*. *glabrata* by approximately 25% (Fig. 4c). These findings show that NETs exhibit antifungal activity against *C*. *glabrata*. Similar inhibition of the biofilm and planktonic *C*. *glabrata* indicates that the biofilm mode of growth does not provide resistance to killing by NETs. Instead, dampened or delayed triggering of NETs appears to account for the resistance of *C*. *glabrata* biofilms to neutrophil killing.

### The mechanisms of NET release for biofilm and planktonic C. *glabrata*

**Figure 5:**
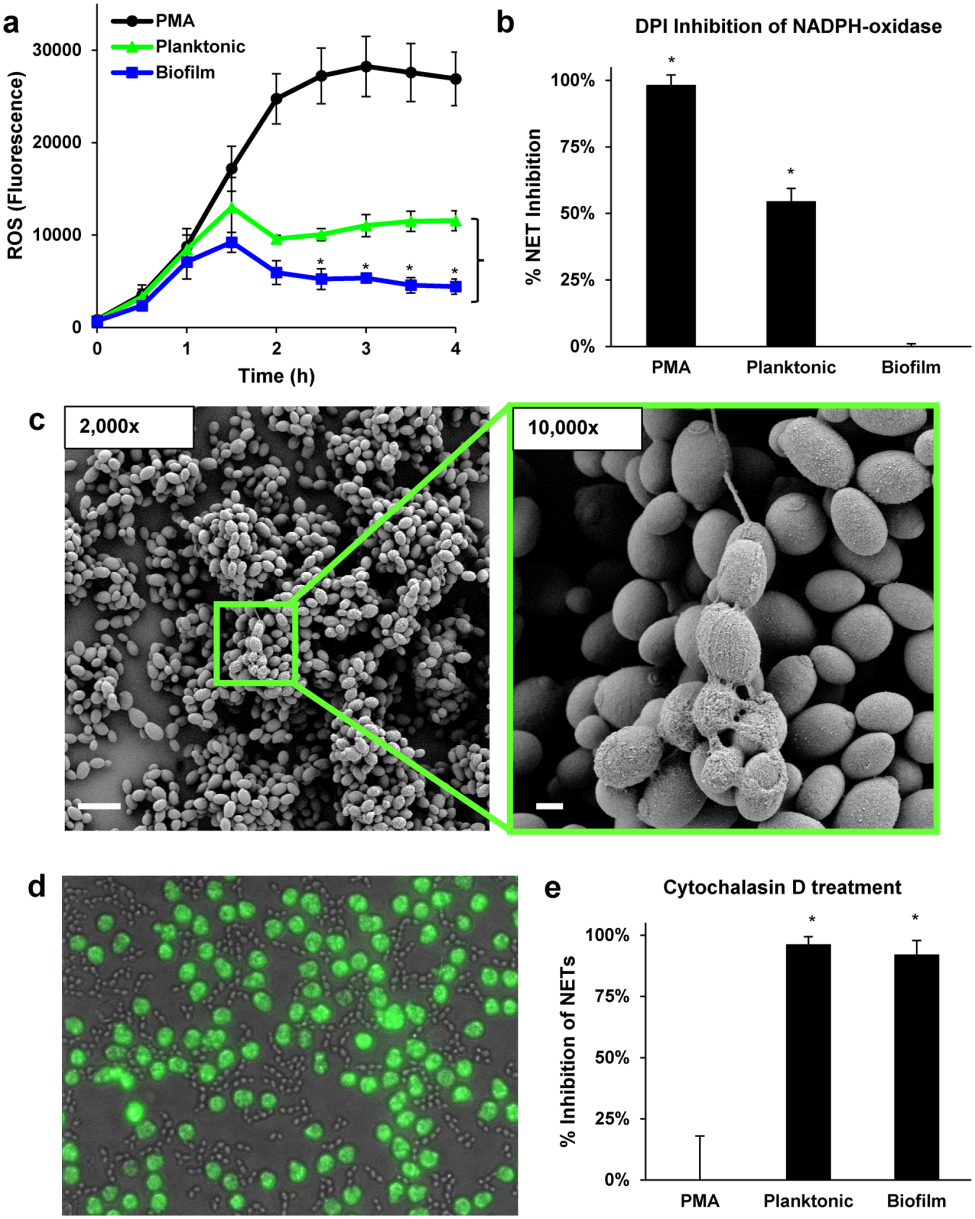
Mechanism of NET induction by planktonic and biofilm *C*. *glabrata*. (**a**) Production of ROS in response to *C*. *glabrata* was measured by fluorescence after neutrophils were pre-stained with oxidative stress indicator CM-H2DCFDA and co-cultured with *C*. *glabrata* over 4 h. The mean and SEM of 4 experiments performed in triplicate is shown. The statistical significance neutrophil production of ROS in response to biofilm and planktonic *C*. *glabrata* were calculated for each time point using a two-tailed Student’s t-test assuming unequal variances, *P < *0*.*05*, *SEM shown*. (**b**) Neutrophils were treated with DPI to inhibit NADPH-oxidase and the release of NETs in response to *C*. *glabrata* was measured by Sytox Green. The percent of the total free DNA (untreated) reduced by DPI-treatment for each condition is shown. Data represent 5 experiments performed in triplicate. Statistical significance was determined using a Student’s t-test compared to no inhibition, *P < *0*.*05*, *SEM shown*. (**c**) Neutrophil-*C*. *glabrata* interactions after 4 h were imaged with scanning electron microscopy. (**d**) Calcein AM-labeled neutrophils (green) were treated with cytochalasin D to inhibit phagocytosis and added to planktonic *C*. *glabrata*. Neutrophil interactions were imaged at 1 h (40x). (**e**) Neutrophils were treated with cytochalasin D to inhibit phagocytosis and the release of NETs in response to *C*. *glabrata* was measured by Sytox Green. The percent of the total free DNA (untreated) reduced by DPI treatment for each condition is shown. Data represent 5 experiments performed in triplicate. Statistical significance was determined using a Student’s t-test compared to no inhibition, *P < *0*.*05*, *SEM shown*.

The difference in triggering of NETs by planktonic and biofilm *C*. *glabrata* prompted exploration of the mechanism unpinning NET release to this pathogen. Prior investigations examining NET formation in response to *C*. *albicans* have identified the involvement of both ROS-dependent and -independent pathways^34,40,46,47^. The generation of ROS by neutrophils exposed to planktonic *C*. *glabrata* suggested the involvement of a ROS-dependent pathway of NET induction (Fig. 2a). A comparison of the neutrophil response to *C*. *glabrata* demonstrated heightened ROS production to planktonic cells in comparison to biofilm, which generated levels near the baseline (Fig. 5a). However, ROS levels were significantly lower upon exposure to *C*. *glabrata*, when compared to PMA treatment. To test the dependence of NET release on ROS, we utilized DPI (diphenylene iodonium), a pharmacological inhibitor of NADPH oxidase^38^. Similar to prior investigations, PMA-induction of NETs, which is ROS-dependent, was abolished by treatment with DPI^38^ (Fig. 5b). In contrast, DPI treatment decreased NET production in response to planktonic *C*. *glabrata* by approximately 50%, consistent with only a partial dependence on ROS (Fig. 5b). As expected, inhibition of NADPH-oxidase had no impact on biofilm-induced NETs, as *C*. *glabrata* biofilms elicited minimal ROS, a phenomenon previously described for biofilms formed by *C*. *albicans* ^12^. These findings suggests the involvement of an alternative, ROS-independent pathway triggering NETs in response to *C*. *glabrata*.

To gain insight into alternative pathways promoting NET release, we utilized time-lapse imaging to visualize neutrophil-*C*. *glabrata* interactions. Imaging of early neutrophil interactions (30–60 min) revealed the rapid engulfment of *C*. *glabrata* by neutrophils pre-stained with Calcein AM (green), often of many yeast per cell (Supplementary Video 1). The ingestion of numerous yeast per phagocyte is similar to prior observations of macrophage-*C*. *glabrata* interactions^48,49^. The findings also corroborate our scanning electron microscopy imaging studies at 30 min, which illustrate the acquisition of numerous yeast per neutrophil (Fig. 2c). For examination of later interactions (2.5–3 h) we included the free DNA stain, propidium iodide (red)^12^. After 2.5 h, many of the neutrophils appeared viable, retaining the Calcein AM stain and excluding propidium iodide (Supplementary Video 2 and Supplementary Fig. 5). However, a subset of the neutrophils had lost this viability staining and exhibited propidium iodide staining, often displaying a slight halo, suggesting NET release. Time-lapse imaging revealed continued phagocytosis of *C*. *glabrata*. Nearly all of the *C*. *glabrata* remained viable, marked by exclusion of propidium iodide, with the exception of several propidium iodide-stained groups of *C*. *glabrata*. Interestingly, these appeared to be extruded from neutrophils, remaining adherent by only a tether. On several occasions, the bundles of yeast moved in concert with the associated neutrophil during migration (Supplementary Video 3). A similar release of inviable *C*. *glabrata* has previously been described for neutrophils and termed “dumping”^50^. However, in our studies, the propidium iodide staining of the *C*. *glabrata* groups was more diffuse than would be expected for individual inviable cells, prompting the question of NET encasement accounting for the fluorescence. By scanning electron microscopy, we identified comparable groups of encased *C*. *glabrata* with a single fibrillary extension (Fig. 5c). The web-like covering of these cell groups was consistent with the release of NETs.

We next questioned a role for phagocytosis in the pathway triggering NET release to *C*. *glabrata*. To determine if phagocytosis was a prerequisite for NET release, we pre-treated neutrophils with cytochalasin D, which impaired the phagocytosis of *C*. *glabrata* (Fig. 5d and Supplementary Video 4). While cytochalasin D treatment had no impact on PMA-induced NET release, it eliminated NET formation in response to both planktonic and biofilm *C*. *glabrata* (Fig. 5e). Together, the findings show that *C*. *glabrata* induces NET release through a phagocytosis-dependent pathway distinct from the mechanism of PMA-induction.

### Neutrophil response to *C*. *glabrata in vivo*

**Figure 6:**
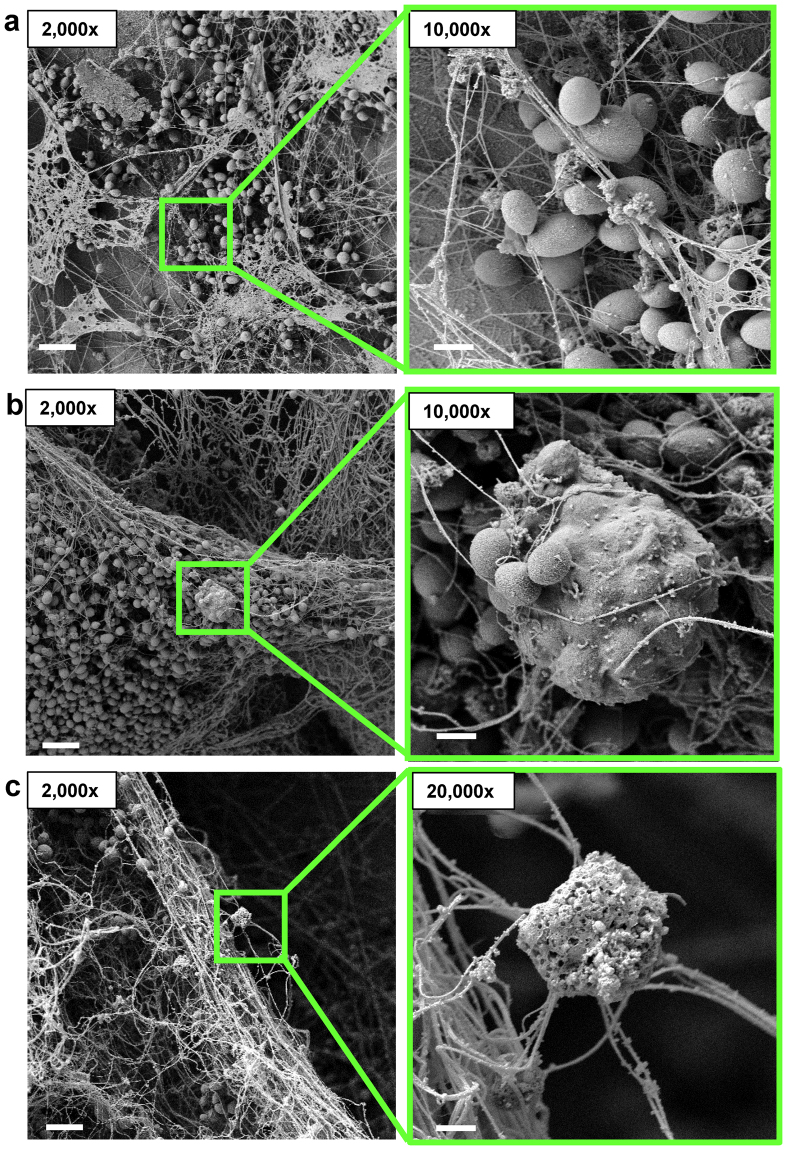
*C*. *glabrata* appears to induce the formation of NETs *in vivo*. (**a**,**b**,**c**) *C*. *glabrata* was inoculated in the lumen of rat jugular catheters. After 48 h, catheters were harvested and host-fungal interactions on the luminal catheter surface were observed by scanning electron microscopy. Measurement bars represent 10 µm and 1 µm for 2,000x and 10,000x images, respectively.

To investigate the neutrophil response to *C*. *glabrata in vivo*, we selected a rat venous catheter model of infection^35,51,52^. In this model, *C*. *glabrata* grows adherent to the luminal surface of a vascular catheter, allowing for the imaging of host-*Candida* interactions upon catheter removal and processing^35^. After 48 h, imaging of the catheters by scanning electron microscopy revealed a heterogeneous biofilm of adherent yeast cells encased in extracellular material and thread-like structures coating the biofilm (Fig. 6a). Host cells, including leukocytes, were observed to be interacting with *C*. *glabrata*. Several of them had engulfed numerous yeast (Fig. 6b), mimicking the initial neutrophil-*C*. *glabrata* interactions observed in vitro (Fig. 2c and Supplementary Video 1). Other cells were extending fibrils consistent with NET release (Fig. 6c). Unlike time course experiments in vitro, the *in vivo* model provides a continuous supply of neutrophils, likely accounting for the multiple interactions observed at a single time point. These findings corroborate our in vitro studies and support a role for NET release in response to *C*. *glabrata* during infection.

## Discussion

Recent investigations have shed light on the importance of NET release for control of candidiasis and other invasive fungal infections^22,53–56^. This neutrophil effector mechanism delivers antimicrobial proteins and peptides while preventing dissemination of infection^19,21^. NET-associated calprotectin has been shown to be critical for the killing of *C*. *albicans* in vitro and for the clearance of candidiasis in murine models of infection^22^. As the larger size of the hyphae produced by *C*. *albicans* precludes phagocytosis by neutrophils, NET-mediated fungal killing is thought to be crucial for control of invasive candidiasis^20^.

We have shown that *C*. *glabrata* induces the release of NETs. The process involves the phagocytosis of yeast, often multiple per neutrophil, followed by the extrusion of DNA with citrullinated histones and neutrophil death. As *C*. *glabrata* does not form hyphae, this is a surprising finding. For *C*. *albicans*, hyphae appear to trigger a NET response more robust than yeast morphotypes^20,34,40^. While yeast forms of *C*. *albicans* have been shown to induce NETosis, these studies have primarily examined NET formation in the context of assay conditions promoting the induction of filamentous growth^12,19^. However, an investigation employing a yeast-locked mutant revealed neutrophil phagocytosis of yeast cells without the generation of NETs^20^. An additional study demonstrating diminished NET release to yeast during non-hyphal inducing assay conditions further supports the importance of filamentous growth to NET release against *C*. *albicans* ^34^. Therefore, the induction of NETs by the yeast forms of *C*. *glabrata* signifies a neutrophil response distinct from *C*. *albicans* and highlights the importance of examining individual species in host-pathogen interactions.

The current studies show that biofilms formed by *C*. *glabrata* resist killing by neutrophils (Fig. 4a). This mechanism of resistance to immune attack has been described for other *Candida* spp., including *C*. *albicans* and *C*. *parapsilosis*, with biofilms exhibiting 5-fold increase in resistance to neutrophil killing^9,11,12,45^. For *C*. *albicans*, biofilms drastically impair the release of NETs, permitting fungal survival, through a mechanism linked to production of an extracellular matrix^12,57^. The composition of the biofilm extracellular matrix, the material first encountered by immune cells, has been well described for *C*. *albicans* ^58,59^. Polysaccharides unique to the biofilm matrix and residing in a mannan-glucan complex have been linked to the impaired release of NETs^12,59^. Like *C*. *albicans*, *C*. *glabrata* biofilm formation involves production of an extracellular matrix rich in polysaccharides^26,60^. However, it is not known if these polysaccharides exist in a mannan-glucan complex similar to that observed for *C*. *albicans*. Therefore, it is somewhat surprising that biofilm formation by *C*. *glabrata* modified NET formation, suggesting an inhibitory pathway conserved among the *Candida* biofilms. However, in contrast to observations for *C*. *albicans*, the current investigation shows that *C*. *glabrata* biofilms permit a degree of NET release, although to a lesser extent than planktonic *C*. *glabrata* (Fig. 4b). Differences in extracellular matrix and/or biofilm architecture may underpin the altered NET release in response to *C*. *albicans* and *C*. *glabrata* biofilms.

It has become increasingly clear that multiple pathways are capable of triggering NET production and that these pathways vary with respect to their dependence on ROS, involvement of histone citrullination, and timing of NET release^34,38–43^. However, less is known about how activation of these specific pathways influences immunity and microbial killing. NET release triggered by planktonic *C*. *glabrata* exhibited features of both classical (ROS-dependent) and rapid (ROS-independent) pathways, similar to that observed for *C*. *albicans* ^34,40,52–54^. In contrast, NET formation in response to *C*. *glabrata* biofilm was delayed. It is interesting to speculate a role for delayed NET production as a microbial survival mechanism. Unlike biofilms formed by the fungal pathogen *Aspergillus fumigatus*, which resist killing by NETs, *C*. *glabrata* biofilms were susceptible to pre-formed NETs, similar to the pattern observed for *C*. *albicans* biofilms^12,55^. These results point to impairment of neutrophil function as a common feature of *Candida* biofilms to avoid neutrophil attack, conserved across species.

## Methods

### Organisms and inoculum

*C*. *glabrata* isolates CG 005, CG 006, CG 5376 and ATCC 2001 were used in this study. The previously described strains CG 5376 and ATCC 2001 were initially isolated from the bloodstream and gastrointestinal tract, respectively^35,36^. CG005 and CG006 strains are vaginal isolates newly described in this study and were obtained in accordance with relevant guidelines and regulations, including the Policy for the Protection of Human Subjects. Strain speciation was confirmed for all isolates by PCR, as previously described^61^. Strains were stored in 15% (vol/vol) glycerol stock at −80 °C and maintained on yeast extract-peptone-dextrose (YPD) medium + uridine (1% yeast extract, 2% peptone, 2% dextrose, and 80 μg/ml uridine) prior to experiments. Cultures were propagated overnight in YPD + uridine at 30 °C on an orbital shaker at 200 RPM. For studies using planktonic organisms, overnight cultures were inoculated in YPD + uridine broth and grown at 30 °C for 2 h on an orbital shaker at 200 RPM, washed twice with Dulbecco’s phosphate buffered saline (-calcium, -magnesium) (DPBS) (Hyclone Laboratories Inc., Logan, UT), and enumerated by hemocytometer.

### Human neutrophil collection

Blood was obtained from study participants after informed consent through a protocol approved by the University of Wisconsin Internal Review Board (IRB) in accordance with relevant guidelines and regulations, including the Policy for the Protection of Human Subjects. Peripheral blood neutrophils were purified by negative antibody selection using the MACSxpress Neutrophil Isolation and MACSxpress Erythrocyte Depletion kits (Miltenyi Biotec Inc., Auburn, CA)^12^. Neutrophils were enumerated by hemocytometer and resuspended in RPMI 1640 (without phenol red) supplemented with 2% heat-inactivated fetal bovine serum (FBS) and supplemented with glutamine (0.3 mg/ml) for all experiments. Incubations were at 37 °C with 5% CO_2_.

### *In vitro* microtiter plate biofilms and XTT assay

Biofilms were formed in 96-well plates as previously described^12,62^. Briefly, *C*. *glabrata* was resuspended in RPMI-MOPS at a concentration of 1.5 × 10^6^ cell/ml and 200 µl was added to each well and plates were incubated for 24 h at 37 °C. Biofilm burden was estimated using an XTT (2,3-Bis-(2-Methoxy-4-Nitro-5-Sulfophenyl)-2H-Tetrazolium-5-Carboxanilide) assay as previously described, but with the electron-coupling agent menadione^62^. One hundred µl of the XTT working solution (0.75 mg/ml XTT in PBS with 10 µM menadione (from 10 mM stock in acetone)) was added to each well. After a 30 min incubation, samples were transferred to a Falcon 96 well U bottom plate and centrifuged for 3 minutes at 1,200 × g to pellet cells. Supernatants (110 µl) were collected and transferred to a 96-well flat bottom plate for absorption reading at 492 nm. To determine an equivalent burden of planktonic organisms, biofilm XTT values were compared to XTT values obtained for a dilution series of planktonic organisms. Results showed a burden of approximately 3 × 10^6^ planktonic cells/well to be similar to the biofilm burden (Supplementary Fig. 1). Therefore, this number of planktonic cells was used in neutrophil co-culture experiments comparing their response to biofilm and planktonic cells.

### Biofilm coverslip model

For scanning electron microscopy imaging, a coverslip model of biofilm formation was utilized^12,63^. Briefly, *C*. *glabrata* resuspended in RPMI-MOPS at 1.5 × 10^6^ cells/ml was added to poly-L-lysine coated coverslips (13 mm, Thermanox plastic for cell culture) and allowed to adhere for 30 min at 30 °C. After removal of media and non-adherent cells, 1 ml of RPMI-MOPS was added. Biofilms were propagated for 24 h at 37 °C and washed with DPBS. Neutrophils (5 × 10^5^) were added to biofilm coverslips for 30 min, 1 h, or 4 h, washed gently with DPBS, and prepared for scanning electron microscopy, as described below. For studies utilizing planktonic organisms, a similar burden of planktonic organism was added to the coverslip prior to the addition of neutrophils.

### Sytox Green assays

As an estimate of NET release, we adapted a Sytox Green assay for use with *C*. *glabrata* ^12,37^. *C*. *glabrata* biofilms were grown in wells of 96-well opaque plates, as described above, and neutrophils (2 × 10^5^ cell/well) were added. Following a 4 h incubation, Sytox Green (Life Technologies, Eugene, OR) was added at a final concentration of 1 μM and fluorescence (excitation 500 nm/emission 528 nm) was measured in an automated plate reader. Experiments using planktonic cells were similarly performed by adding and allowing 3 × 10^6^ cells/well to settle prior to the addition of neutrophils. DPI (10 μM) or Cytochalasin D (10 μg/ml) were included to inhibit ROS and phagocytosis, respectively. PMA (100 nM) was included as a positive control and used for normalization between donors. Background fluorescence for each condition was subtracted from total fluorescence values.

### Measurement of ROS

For measurement of neutrophil ROS production, we utilized an oxidative stress assay, as previously described^12^. Briefly, neutrophils were pre-stained with CM-H2DCFDA (Life Technologies, Eugene, OR) in DPBS for 10 min at room temperature in the dark. Neutrophils (2 × 10^5^ neutrophils/well) were added to *C*. *glabrata* biofilms growing in 96-well opaque plates or planktonic *C*. *glabrata* (3 × 10^6^ cells/well). Fluorescence (excitation 495 nm; emission 527 nm) was recorded every 30 min for 4 h. Background fluorescence was determined for each *C*. *glabrata* condition and subtracted from total fluorescence values prior to data analysis. PMA (100 nM) was included as a positive control.

### Fluorescent imaging

Time-lapse fluorescent imaging experiments were performed as previously described^12^. *C*. *glabrata* (5 µl at 3 × 10^7^ cells/ml in DPBS) was added to 384 well plate (Corning 3985) containing 40 µl of media. Neutrophils were labeled with Calcein AM (ThermoFischer Scientific, Waltham, MA) at 0.5 μg/ml in DPBS for 10 min at room temperature in the dark, rinsed twice with DPBS and 5 µl was added to *C*. *glabrata* at a concentration of 2 × 10^5^ cells/ml. For a subset of experiments, 2 µl of propidium iodide (80 µM) was added to wells 15 min prior to imaging and plates were reincubated. Images were obtained every 60 sec using brightfield and fluorescence (excitation 480 nm, emission 525 nm and excitation 565, emission 620) at 20x or 40x for 30 minutes on an inverted microscope (Nikon Eclipse TE300) equipped with a motorized stage (Ludl Electronic Products), charge-coupled device camera (CoolSNAP ES2), and MetaVue imaging software v6.2. Images and videos were compiled using ImageJ. Video is shown at 5 frames per second.

### Immunofluorescent imaging

As a qualitative measure of NETs, immunofluorescent imaging for histone citrullination was performed. *C*. *glabrata* (100 µl at a concentration of 6 × 10^7^ cells/ml) were added to a 4 well µ-Slide (Ibidi) with neutrophils (200 µl at a concentration of 2 × 10^6^ cells/ml). After 4 h, mixtures were fixed with 4% formaldehyde in DPBS for an additional 2 h. Fixed co-cultures were treated with Sytox Green 1 µM in DPBS for 10 min, rinsed 3 × 5 min with DPBS, and incubated with antibody blocking buffer (2% w/v bovine serum albumin (BSA) and 0.02% v/v Tween 20 in DPBS) overnight at 4 °C. All steps were performed very gently to preserve NETs. Following rinsing with antibody binding buffer (0.1% BSA w/v and 0.005% v/v Tween 20 in DPBS), primary antibody (anti-histone H4, citrulline3) in antibody binding buffer at 1:500 was added for 2 h at room temperature^39^. Samples were rinsed gently 3 × 5 min and secondary antibody (goat-anti rabbit IgG Fc DyLight 594 conjugated) at 1:200 in antibody binding buffer was added for a 2 h incubation in the dark. Samples were rinsed 3 × 5 min with antibody binding buffer and brightfield and fluorescent (excitation 480 nm, emission 525 nm and excitation 565, emission 620) images were obtained using the 20x objective on an inverted microscope (Nikon Eclipse TE300) equipped with a charge-coupled device camera (CoolSNAP ES2) and MetaVue imaging software v6.2. Images were processed using ImageJ.

### Killing assays

Briefly, an XTT (2,3-bis-(2-methoxy-4-nitro-5-sulfophenyl)-2H-tetrazolium-5-carboxanilide) metabolic assay was adapted to estimate *C*. *glabrata* viability following co-culture with neutrophils^8,12^. Following a 6, 12, or 24 h incubation period, biofilms grown in microtiter plates were washed with DPBS. In studies comparing the killing of 24 h biofilm and planktonic *C*. *glabrata*, biofilms were compared to planktonic cells at a concentration of 3 × 10^6^ cells/well. Neutrophils were added to a final concentration of 1.5 × 10^6^ cells/well (effector:target of 1:2). Following a 4 h co-incubation, an XTT assay was performed, as described above. To determine percent killing, values were compared to wells without neutrophils after subtraction of neutrophil generated XTT baseline absorbance, which was less than 5% of the total read. For killing assays with preformed, PMA-induced NETs, neutrophils at 1.5 × 10^7^ cells/ml were incubated with or without 100 nM PMA for 90 min at 37 °C with gentle rotation. After 90 min, PMA-induced NETs or neutrophil controls were added to biofilms or planktonic cells, as above. After a 10 min incubation, an XTT assay was performed.

### *In vivo* venous catheter biofilm model

All animal procedures were approved by the Institutional Animal Care and Use Committee at the University of Wisconsin according to the guidelines of the Animal Welfare Act, and The Institute of Laboratory Animal Resources Guide for the Care and Use of Laboratory Animals. Specific-pathogen-free rats (Harlan Sprague-Dawley, Indianapolis, Ind.) were used for all studies. A jugular vein rat central venous catheter biofilm infection model was used as previously described^35,52^. Briefly, 24 h following surgical jugular venous catheter insertion, *C*. *glabrata* at 10^6^ cells/ml was instilled in the catheter lumen and flushed at 6 h. After a 48 h incubation, the catheters were harvested and collected for imaging by scanning electron microscopy, as described below.

### Scanning electron microscopy

Rat vascular catheter and coverslips were processed and imaged by scanning electron microscopy, as previously described^12,52^. Briefly, after washing with DPBS, specimens were placed in fixative (4% formaldehyde, 1% glutaraldehyde, in PBS) overnight. After washing with PBS, they were treated with 1% osmium tetroxide and washed again with PBS. Samples were dehydrated through a series of ethanol washes followed by critical point drying and mounted on aluminum stubs with carbon conductive adhesive tabs. Following sputter coating with platinum, samples were imaged in a scanning electron microscope (LEO 1530) at 3 kV.

### Statistics

Experiments were performed at least 3 times using neutrophils from different donors on different days. Statistical analyses were performed by Student’s t-test (two-tailed) or ANOVA with comparisons by the Holm-Sidak method using Sigma Stat or Excel software. Differences of P < 0.05 were considered significant.

### Data availability

The data that support the findings of this study are available from the corresponding author upon request.

## Electronic supplementary material

Video 1

Video 2

Video 3

Video 4

Supplementary Information
