# Supplementary material for: Mechanisms involved in the triggering of neutrophil extracellular traps (NETs) by Candida glabrata during planktonic and biofilm growth
Source: Sci Rep. 2017 Oct 12;7:13065. doi: 10.1038/s41598-017-13588-6 (PMC5638821; doi:10.1038/s41598-017-13588-6)
Supplement: Supplementary file 5 — Supplementary Information [file 41598_2017_13588_MOESM5_ESM.pdf]

**Supplementary Information**

# Mechanisms involved in the triggering of neutrophil extracellular traps (NETs) by *Candida glabrata* during planktonic and biofilm growth.

Chad J. Johnson[1], John F. Kernien[1], Amanda R. Hoyer[1], and Jeniel E. Nett[1,2*]

[1]Department of Medicine, University of Wisconsin, Madison, WI, United States of America

[2]Department of Medical Microbiology and Immunology, University of Wisconsin, Madison, WI, United States of America

[*]Corresponding author

jenett@medicine.wisc.edu

**Supplementary Video 1. Time-lapse imaging of neutrophil-*C. glabrata* interactions (30 min).** Calcein AM-labeled neutrophils (green) were added to *C. glabrata*. After a 30 min incubation, neutrophil interactions were imaged with time lapse at 20x. Brightfield and fluorescent images were then collected every 1 min for 1 h and compiled at 5 frames per second.

**Supplementary Video 2. Time-lapse imaging of neutrophil-*C. glabrata* interactions (2.5 h).** Calcein AM-labeled neutrophils (green) were added to *C. glabrata* and co-cultured for 2 h 30 min. Free DNA stain propidium iodide (red) was added prior to imaging at 2.5-3 h at 20x. Brightfield and fluorescent images were then collected every 1 min for 1 h and compiled at 5 frames per second.

**Supplementary Video 3. Neutrophils release tethered groups of *C. glabrata*.** As in Video 2, Calcein AM-labeled neutrophils (green) were added planktonic *C. glabrata* and co-cultured for 2 h 30 min. Free DNA stain propidium iodide (red) was added prior to imaging at 2.5-3 h at 20x. To more closely view the movement of propidium idodide-stained *C. glabrata*, images from Video 2 are shown with fluorescent channels only. Arrows show groups of propidium iodide-labeled *C. glabrata* at the start of the image series.

**Supplementary Video 4. Cytochalasin D inhibits phagocytosis of *C. glabrata*.** Calcein AM-labeled neutrophils (green) were treated with cytochalasin D and added to planktonic *C. glabrata*. Neutrophil interactions were imaged with time-lapse for 1h at

40x. Brightfield and fluorescent images were then collected every 1 min for 1 h and compiled at 5 frames per second.

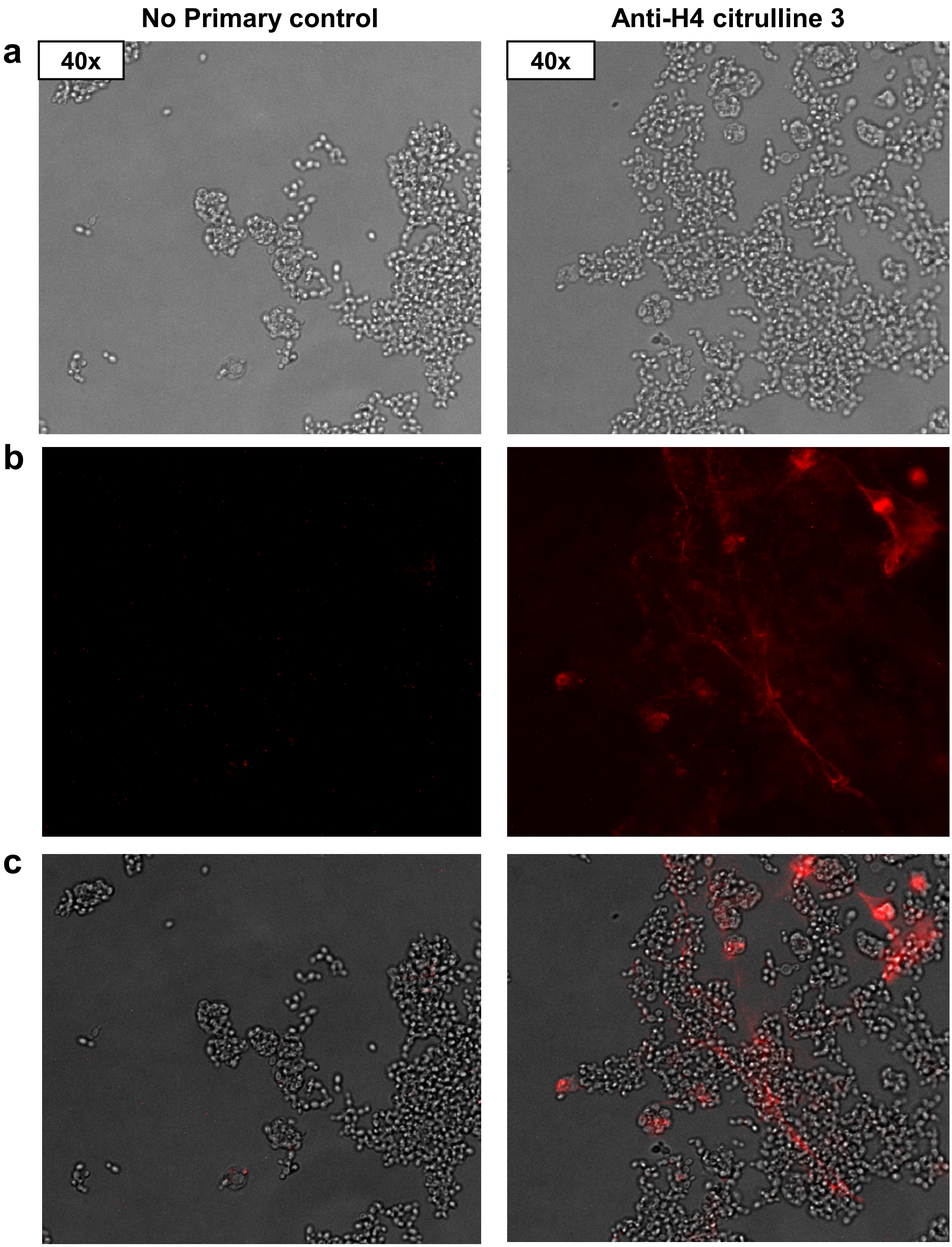


**Supplementary Figure 1.** Following co-culture with *C. glabrata* for 4 h, samples were immunolabeled with an anti-citrullinated H4 antibody and a fluorescently labeled (DyLight) secondary antibody. The secondary antibody alone control is shown on the left. Brightfield (**a**) and fluorescent 565/620 nm (**b**) images are merged in **c**.

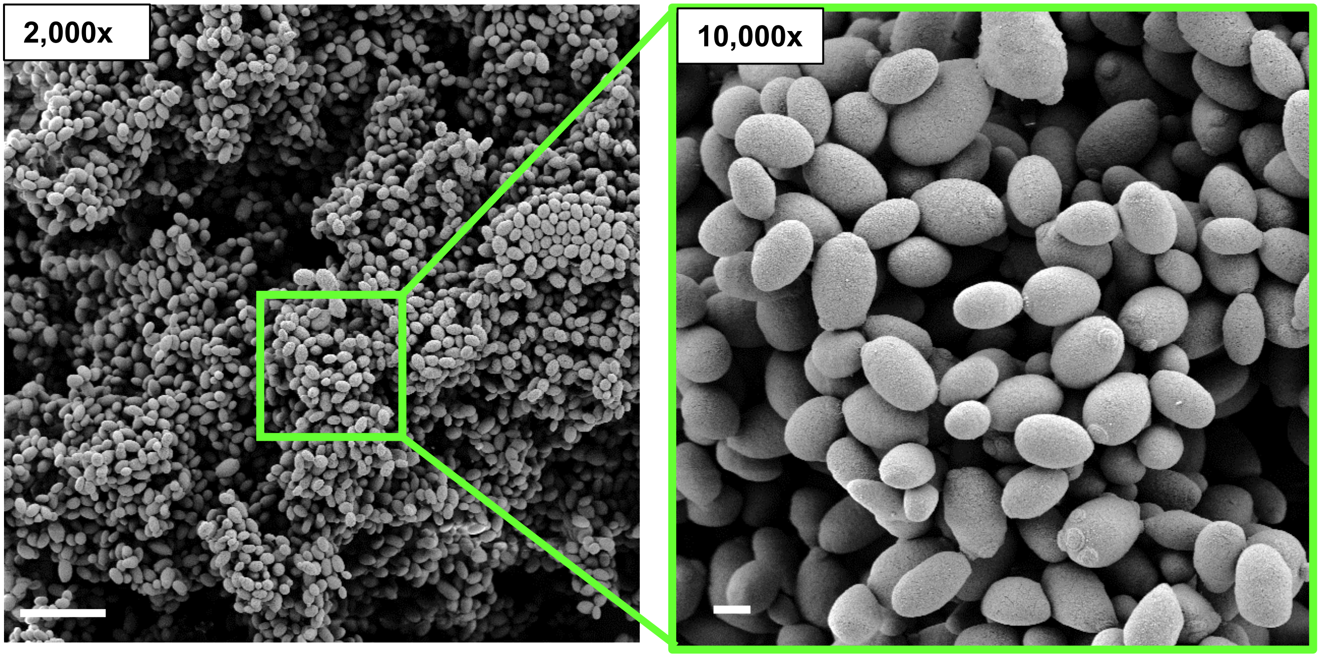


**Supplementary Figure 2**. ***C. glabrata* forms biofilms are composed of yeast.** *C. glabrata* (CG 006) biofilms were grown on coverslips for 24 h and imaged by scanning electron microscopy. Measurement bars represent 10 μm and 1 μm for 2,000x and 10,000x images, respectively.

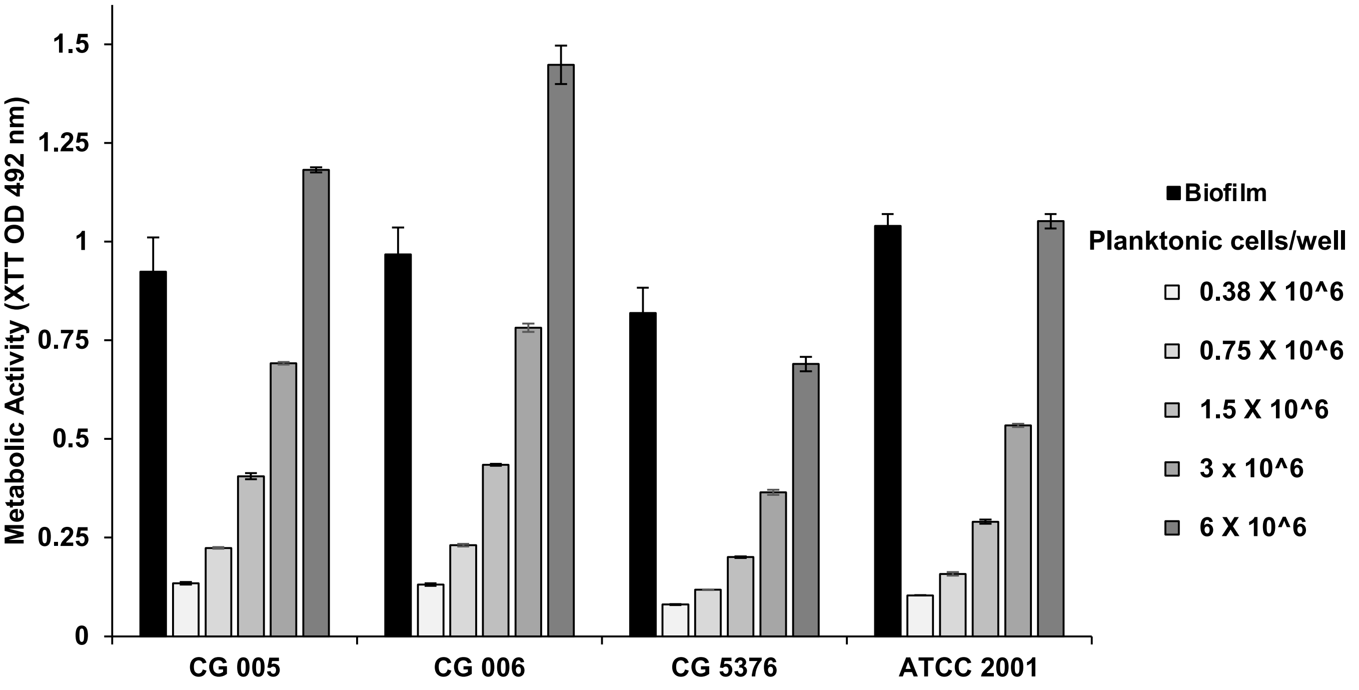


**Supplementary Figure 3. Biofilm and planktonic normalization**. Biofilm formation after 24 h was measured using XTT assays. The metabolic activities of biofilms were compared to various concentrations of planktonic cells to determine a similar burden for studies. Assays were performed in triplicate on 3 occasions and mean values of a representative experiment are shown. Error bars represent SD.

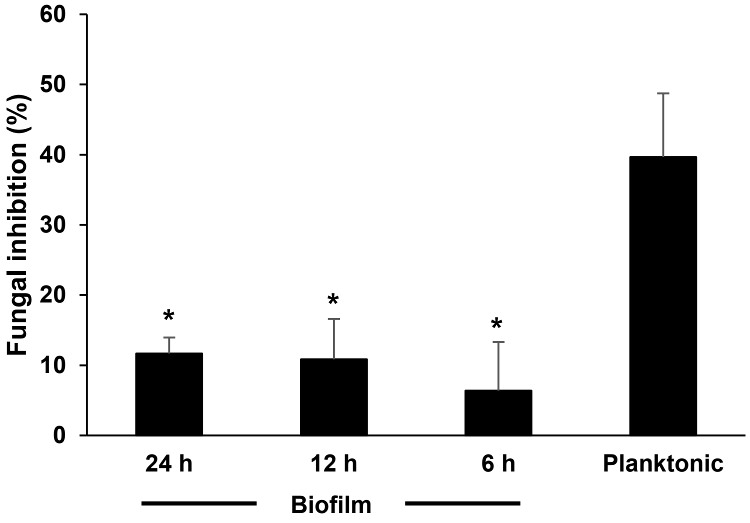


**Supplementary Figure 4. Neutrophil inhibition of *C. glabrata* during stages of biofilm formation and planktonic growth.** Planktonic and biofilm *C. glabrata* were co-cultured with human neutrophils for 4 h and fungal inhibition was estimated by an XTT assay. Results were normalized to the no neutrophil controls, and data from 3 experiments performed in triplicate were combined. Statistical significance was determined using a two-tailed Student's t-test assuming unequal variances, *$P<0.05$ for biofilm condition compared to planktonic control, SEM shown.

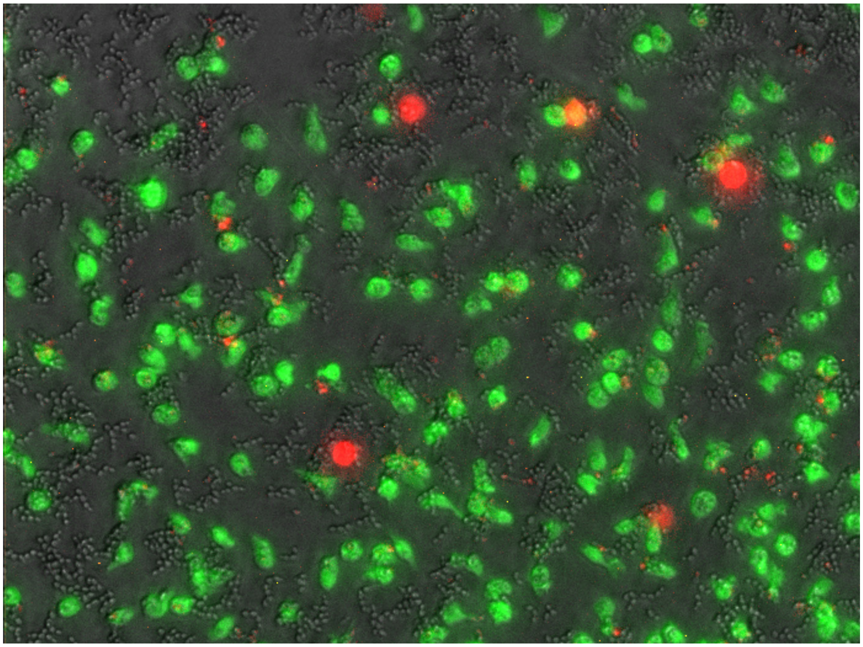

**Supplementary Figure 5. Neutrophil-*C. glabrata* interactions at 2.5 h.** Calcein AM-labeled neutrophils (green) were added to *C. glabrata* and co-cultured for 2 h 30 min. Free DNA stain propidium iodide (red) was added prior to imaging at 20x.
